# Supplementary material for: ProteinShader: illustrative rendering of macromolecules
Source: BMC Struct Biol. 2009 Mar 30;9:19. doi: 10.1186/1472-6807-9-19 (PMC2672931; doi:10.1186/1472-6807-9-19)
Supplement: Additional file 1 — ProteinShader program without source code. This compressed file contains the complete ProteinShader program including associated libraries, but no source code. A README.txt file gives an overview of the ProteinShader distribution, and the index.html file in the help subdirectory has directions on getting started with the program as well as a set of tutorials. [file 1472-6807-9-19-S1.zip › ProteinShader-beta-0_9_4-binary/help/api/org/proteinshader/gui/enums/StyleEnum.html]

StyleEnum (ProteinShader API)


|  |  |  |
| --- | --- | --- |
| **Overview** \| **Package** \| **Class** \| **Use** \| **Tree** \| **Deprecated** \| **Index** \| **Help** | |  |
| **PREV CLASS**   NEXT CLASS | **FRAMES**    **NO FRAMES**     **All Classes** |  |
| SUMMARY: NESTED \| ENUM CONSTANTS \| FIELD \| METHOD | DETAIL: ENUM CONSTANTS \| FIELD \| METHOD |  |


---


## org.proteinshader.gui.enums Enum StyleEnum

```
java.lang.Object
  java.lang.Enum<StyleEnum>
      org.proteinshader.gui.enums.StyleEnum
```

**All Implemented Interfaces:**: Serializable, Comparable<StyleEnum>

---

``` public enum StyleEnum extends Enum<StyleEnum> ```

Provides an enumeration to specify what style should be used to display a
Structure: Cartoon (Tubes and Ribbons) or Atom (Spheres and Cylinders).

---

**Enum Constant Summary**

`BALLS_AND_STICKS`
  
          Show the molecule using a balls-and-sticks representation.

`FRENET_FRAMES`
  
          Show the local coordinate frames of the tube segments.

`RIBBONS`
  
          Show the molecule as three-dimensional ribbons.

`RIBBONS_AND_TUBES`
  
          Show the molecule as three-dimensional ribbons (beta-strands)
and tubes (alpha-helices).

`SPACE_FILLING`
  
          Show the molecule using space filling representation (spheres
only).

`STICKS`
  
          Show the molecule using sticks (thin cylinders) only.

`TUBES`
  
          Show the molecule as three-dimensional tubes.


| **Method Summary** | |
| --- | --- |
| `static StyleEnum` | `getDefaultStyle()`             Returns the default style, which is BALLS\_AND\_STICKS. |
| `String` | `getMenuName()`             Returns the name of the StyleEnum in a form suitable for use in a menu. |
| `String` | `toString()`             Returns the name of the StyleEnum in a form suitable for use in a menu. |
| `static StyleEnum` | `valueOf(String name)`             Returns the enum constant of this type with the specified name. |
| `static StyleEnum` | `valueOfMenuName(String menuName)`             Returns the StyleEnum with the same menu name as the String given as an argument. |
| `static StyleEnum[]` | `values()`             Returns an array containing the constants of this enum type, in the order they're declared. |

**Methods inherited from class java.lang.Enum**

`clone, compareTo, equals, getDeclaringClass, hashCode, name, ordinal, valueOf`

**Methods inherited from class java.lang.Object**

`finalize, getClass, notify, notifyAll, wait, wait, wait`

**Enum Constant Detail**

### TUBES

```
public static final StyleEnum TUBES
```

:   Show the molecule as three-dimensional tubes.
    The menu name for this enum is "Tubes".

---


### RIBBONS

```
public static final StyleEnum RIBBONS
```

:   Show the molecule as three-dimensional ribbons.
    The menu name for this enum is "Ribbon".

---


### RIBBONS\_AND\_TUBES

```
public static final StyleEnum RIBBONS_AND_TUBES
```

:   Show the molecule as three-dimensional ribbons (beta-strands)
    and tubes (alpha-helices).
    The menu name for this enum is "Ribbon and Tubes".

---


### FRENET\_FRAMES

```
public static final StyleEnum FRENET_FRAMES
```

:   Show the local coordinate frames of the tube segments.
    The menu name for this enum is "Frenet Frames".

---


### SPACE\_FILLING

```
public static final StyleEnum SPACE_FILLING
```

:   Show the molecule using space filling representation (spheres
    only). The menu name for this enum is "Space Filling".

---


### BALLS\_AND\_STICKS

```
public static final StyleEnum BALLS_AND_STICKS
```

:   Show the molecule using a balls-and-sticks representation.
    The menu name for this enum is "Balls and Sticks".

---


### STICKS

```
public static final StyleEnum STICKS
```

:   Show the molecule using sticks (thin cylinders) only.
    The menu name for this enum is "Sticks".


**Method Detail**

### values

```
public static final StyleEnum[] values()
```

:   Returns an array containing the constants of this enum type, in
    the order they're declared. This method may be used to iterate
    over the constants as follows:

    ```
    for(StyleEnum c : StyleEnum.values())
            System.out.println(c);
    ```

    :   **Returns:**: an array containing the constants of this enum type, in the order they're declared

---


### valueOf

```
public static StyleEnum valueOf(String name)
```

:   Returns the enum constant of this type with the specified name.
    The string must match *exactly* an identifier used to declare an
    enum constant in this type. (Extraneous whitespace characters are
    not permitted.)

    :   **Parameters:**: `name` - the name of the enum constant to be returned. **Returns:**: the enum constant with the specified name **Throws:**: `IllegalArgumentException` - if this enum type has no constant with the specified name

---


### getDefaultStyle

```
public static StyleEnum getDefaultStyle()
```

:   Returns the default style, which is BALLS\_AND\_STICKS.

    :   **Returns:**: The default style.

---


### getMenuName

```
public String getMenuName()
```

:   Returns the name of the StyleEnum in a form suitable for use in
    a menu.

    :   **Returns:**: The StyleEnum as a String.

---


### toString

```
public String toString()
```

:   Returns the name of the StyleEnum in a form suitable for use in
    a menu.

    :   **Overrides:**: `toString` in class `Enum<StyleEnum>`
    :   **Returns:**: The StyleEnum as a String.

---


### valueOfMenuName

```
public static StyleEnum valueOfMenuName(String menuName)
```

:   Returns the StyleEnum with the same menu name as the String
    given as an argument.

    :   **Returns:**: The StyleEnum matching the menu name. **Throws:**: `IllegalArgumentException` - if the menu name does not match a StyleEnum.


---


|  |  |  |
| --- | --- | --- |
| **Overview** \| **Package** \| **Class** \| **Use** \| **Tree** \| **Deprecated** \| **Index** \| **Help** | |  |
| **PREV CLASS**   NEXT CLASS | **FRAMES**    **NO FRAMES**     **All Classes** |  |
| SUMMARY: NESTED \| ENUM CONSTANTS \| FIELD \| METHOD | DETAIL: ENUM CONSTANTS \| FIELD \| METHOD |  |


---

# *Copyright © 2007-2008*
